# Does Speaking the Same Language With the Caretakers Associate With a Higher Neuraxial Labor Analgesia Use Rate?

**DOI:** 10.1111/aas.70248

**Published:** 2026-05-05

**Authors:** Luisa Pirsko, Anna L. U. Väänänen, Riina Jernman, Antti Väänänen

**Affiliations:** ^1^ Department of Anaesthesiology and Intensive Care University of Helsinki and Helsinki University Hospital Helsinki Finland; ^2^ Faculty of Social Sciences University of Helsinki Helsinki Finland; ^3^ Department of Obstetrics and Gynaecology University of Helsinki and Helsinki University Hospital Helsinki Finland

## Abstract

Use of neuraxial analgesia requires communication between the parturient and her caretakers. In this retrospective study, the use of labor analgesia is compared between parturients whose primary language is other than Finnish or Swedish and who don't communicate in these languages or English without an interpreter (Category I), who communicate in Finnish, Swedish, or English (Category II), and primary Finnish or Swedish speakers (Category III). The primary outcome of this study is the association of communication language categories with neuraxial analgesia use. The secondary outcome is the incidence of vaginal delivery without pharmacological pain relief. The parturient and labor parameters (age, body mass index (BMI), primiparity, gestational age, prior consultations for fear‐of‐childbirth (FOC), induction of labor, use of oxytocin, labor analgesia interventions, outcome of the attempted labor) were recorded from the electronic patient database for all 13,707 parturients that attempted vaginal delivery in the Helsinki region delivery hospitals during 2022. The distribution of parturients was 15.3%, 10.3%, and 74.4% in the language categories I, II, and III, respectively. The use rate of neuraxial analgesia was 60.8%, 65.8%, and 75.3% in the categories I, II, and III respectively. After adjustment for primiparity, maternal BMI, gestational age, FOC diagnosis, induction of labor, oxytocin use, episiotomy, and outcome of labor, the adjusted OR (aOR) for neuraxial analgesia use was (1.283 [1.093–1.506]) among parturients in Cat II compared to Cat I. Correspondingly, the ability to communicate directly (Cat II) was associated with lower use of only non‐neuraxial pharmacological analgesia (aOR 0.789 [0.654–0.951]) compared to those incapable of direct communication (Cat I). No association was seen with language capability (Cat II vs. Cat I) and delivery without pharmacological pain relief (any delivery type: aOR 0.815 [0.652–1.019]; vaginal delivery: aOR 0.825 [0.645–1.055]). Ability to communicate directly with the staff is associated with shifting from non‐neuraxial techniques to neuraxial analgesia but not an increase in general labor analgesia use. Cultural trends may be associated with labor analgesia choices more than language capabilities. Promotion of labor analgesia use should go beyond translation services and requires antenatal education of the available options.

**Editorial Comment:**

This study demonstrates that language ability alone does not determine labor analgesia use but instead shapes the selection of analgesia methods. Cultural context and antenatal knowledge appear to play a central role in analgesia decision‐making. Efforts to promote equitable maternity care should therefore extend beyond translation services to include culturally responsive education and communication strategies that support informed maternal choice.

## 1 Introduction

Neuraxial analgesia is a well‐established and highly effective method to control labor pain, resulting in lower pain scores and better satisfaction than systemic pharmacological pain relief methods [1]. Its safe use requires the ability to communicate with the parturient. Since the beginning of the 1990's, a steady flux of immigrants has arrived in Finland, most of whom live in the capital area around Helsinki. Due to the age profile of the immigrant population, a recent sociodemographic study identified that the vast majority of parturients with a foreign background are themselves first‐degree immigrants [2]. Currently, approximately a quarter of the parturients delivering in the Helsinki area delivery hospitals speak a primary language other than Finnish or Swedish, the two official languages in Finland.

Since most of the information available on maternity services, including pain relief during labor, is available in Finnish and Swedish, there may be significant differences in accessibility to information regarding labor analgesia depending on the language skills of the parturient. Prior Scandinavian studies have identified that immigrants from the former Yugoslavia, Middle East, Turkey, and Somalia are less prone to use neuraxial analgesia compared to the parturients born in Sweden or Norway [3, 4, 5]. Meanwhile, neuraxial analgesia use may be more common among the immigrants from European countries, including Finland [3, 5]. This may imply that cultural norms and trends may play a significant role in the use and choice of labor analgesia. The potential reluctance towards neuraxial analgesia use could be affected when the parturient can speak directly with the nursing staff, particularly in a birth setting where the institutional labor analgesia practices rely heavily on neuraxial analgesia techniques. While the association between the maternal country of birth and the use of neuraxial analgesia has been studied in the Scandinavian countries, the association between the potential language barrier and the choice of labor analgesia has not been previously studied [3, 4, 5].

In this retrospective study, we analyze the use of neuraxial and other labor analgesia forms among parturients with different communication languages in a publicly funded delivery healthcare system with a 72% neuraxial analgesia use rate. We compare the use of neuraxial analgesia in parturients whose primary language is other than Finnish or Swedish. These parturients fall into two categories, which are parturients who cannot communicate in Finnish, Swedish, or English without an interpreter, relative, or a support person and the parturients whose primary language is other than Finnish or Swedish, but who communicate in Finnish, Swedish, or English.

The primary outcome of this study is

- The association of parturient's communication language on the neuraxial analgesia use rate.

The secondary outcome is

- The rate of vaginal delivery without pharmacological pain relief.

The parturient's subjective satisfaction on labor analgesia and birthing experience is routinely asked and documented post‐partum as part of quality‐of‐care monitoring. As an exploratory outcome on equity of care, the association of communication language on the rate of documentation of the satisfaction score is assessed alongside the actual scores.

## 2 Materials and Methods

### 2.1 Collection of Data

Data relating to parturients that delivered in any of the Helsinki, Finland, area delivery hospitals between 1 January 2022 and 31 December 2022 were collected. These data included all 13,707 parturients that were admitted to the labor wards for the purpose of giving birth during the time frame of the study irrespective of the actual mode of delivery. Direct admission for a cesarean delivery of any urgency without any documentation regarding an attempt to deliver vaginally was considered an exclusion criterion.

The collected parameters included all information on the parturients' primary language and communication language between February 2020 and the admission for delivery. Background parameters included information on the number of prior deliveries, maternal age, body mass index (BMI), duration of pregnancy in weeks, diagnosis for fear of childbirth (FOC) if set before the date of delivery, any means used to induce labor, use of oxytocin during labor, use of episiotomy, and outcome of labor (spontaneous vaginal, instrumental vaginal, or intrapartum cesarean delivery). All labor analgesia information was retrieved from the birth report forms and regarding neuraxial analgesia procedures, these were verified from the actual given medications. The different forms of neuraxial analgesia used, along with the detailed protocols for neuraxial analgesia, are described in a prior publication from the same database [6]. For this study, neuraxial analgesia was considered used if any form of spinal, epidural, or combined spinal/epidural analgesia was used at the labor ward. The collected data are validated in the delivery unit for reporting to the national birth registry, and the reporting forms were used as the initial source of information. The information is to a large degree mandatory to report.

Data on birthing experience including satisfaction on analgesia during labor is questioned as part of the routine interview on the first or the second day after delivery at the maternity ward. This data is used as quality indicators in‐house and to screen the parturients for a possible post‐natal visit to mitigate the effects of possible poor delivery experience on the requested mode of delivery in future pregnancies. The interviews are done by the postnatal ward midwives who did not participate in the labor itself. Among the questions, the parturients are asked to rate their satisfaction on analgesia on a numeral rating scale (0 = complete dissatisfaction to 10 = excellent satisfaction).

### 2.2 Definition of Communication Language Categories

The patient journaling system (Apotti/Epic systems corporation, USA, introduced in February 2020 in the Helsinki area delivery hospitals) contains two language categories. The primary (first) language of the parturient and the used communication language of the parturient. These can be updated whenever the parturient signs in for an appointment during her contacts with the health care system. Additionally, the system can contain information regarding the need for an interpreter.

For the analysis of data, the communication language was categorized into three different categories:

- The parturient's primary language is other than Finnish or Swedish and the language of communication is other than Finnish, Swedish or English. Additionally, if the parturient is marked to require an interpreter for her appointments with the healthcare, she will be categorized into this category. This category is used as a reference category and these parturients are referred to as non‐directly communicating parturients.
- The parturient's primary language is marked as other than Finnish or Swedish, but the parturient's language of communication is either Finnish, Swedish or English and there is no marking of requiring an interpreter after February 2020 and during the admission for delivery. These parturients are considered able to communicate directly with the staff.
- Primary language and communication language are both marked as either Finnish or Swedish, the official languages of Finland.

This study addresses the association of documented communication language with the outcome measures such as use of labor analgesia. The country of birth, potential immigration status, or ethnic background of the parturients is not documented in the patient journaling system and is not included in the study data.

### 2.3 Statistical Analysis

The categorical data are expressed as numbers and percentages within the communication language category while continuous data are expressed as mean and standard deviation. For the satisfaction on analgesia scores, the prevalence of full satisfaction (10/10) was measured and analyzed as a dichotomous outcome. Analysis for differences between the communication language categories was carried out by chi‐square tests for categorical data and two‐sided *t*‐tests for continuous data and *p* < 0.05 was considered to mark a statistically significant difference.

The association of communication language category on the likelihood of using different forms of labor analgesia was assessed by logistic regression using all other documented parameters as potential confounders. Those potential cofactors showing univariate *p* value of less than 0.200 were taken into the multivariate analysis without further fitting of the model. Unadjusted and adjusted odds ratios are reported along with their 95% confidence intervals.

The parturients were grouped into geographical subgroups according to their primary language (see Table 1). This was necessary to adjust for the different likelihood of being allocated into communication language Category I or II based on approximate geographic area of primary language use.

**TABLE 1:** Parturient communication in Finnish, Swedish or English by geographic language groups of the primary language.

Group	Communication language = Finnish, Swedish or English			Univariate odds ratio
	No, *N* ^a^	Yes, *N* ^b^	All, *N* (%)	For Category II vs. Category I
Finnish/Swedish^c^			10,197 (74.4%)	
Europe (EEA)	383	426	809 (5.9%)	1.922 (1.640–2.253)
Europe (non‐EEA)	495	220	715 (5.2%)	0.594 (0.499–0.701)
Middle East, North Africa	329	148	477 (3.5%)	0.626 (0.509–0.770)
Sub‐Saharan Africa	313	282	595 (4.3%)	1.415 (1.185–1.690)
South Asia	295	137	432 (3.2%)	0.653 (0.527–0.810)
East Asia	214	132	346 (2.5%)	0.896 (0.713–1.126)
Other	65	71	136 (1.0%)	1.648 (1.169–2.323)
Total (% of all parturients)	2094 (15.3%)	1416 (10.3%)	13,707 (100%)	
**Language groups (N for primary language for the three most common languages):**				
**Europe (EAA):** Estonian (293); English (177); Spanish (57); Romanian; French; German; Portugal; Bulgarian; Polish; Latvian; Italian; Hungarian; Lithuanian; Czech; Greek, Dutch; Slovak; Norwegian, Danish				
**Europe (non‐EEA)**: Russian (411); Albanian (179); Turkish (52); Ukrainian; Bosnian; Moldavia; Serbian; Serbo‐Croatian; Macedonian; Tatar; Belarusian				
**Middle East, North Africa:** Arabic (303); Kurdish (120); Farsi/Persian (41); Sorani; Badini; Hebrew; Kurmandzi				
**Sub‐Saharan Africa**: Somalian (407); Tigrinya (35); Amharic (25); Kinyarwanda; Yoruba; Swahili; Igbo; Lingala; Twi; Akan; Hausa; Ewe; Kikuyu; Ganda; Oromo; Tswana; Wolof				
**South Asia**: Nepal (89); Bengali (85); Dari (72); Urdu; Hindi; Tamil; Pashto; Telugu; Punjabi; Marathi; Malayalam; Sinhala; Dhivehi; Oriya; Sindhi				
**East Asia**: Chinese (99); Vietnamese (83); Tagalog (73); Thai; Japanese; Indonesian; Korean; Khmer; Burmese; Malay				
**Other**: Chechen (17); Uzbek (12); Uyghur (11); Armenian; Turkmen; Azerbaijani; Kazakh; Tajik; Non specified language other than Finnish (81)				

^a^ Language Category I: (unable to communicate in Finnish, Swedish or English and/or needs an interpreter).

^b^ Language Category II: (primary language other than Finnish or Swedish; communicates in Finnish, Swedish or English).

^c^ Language Category III: (primary language Finnish or Swedish).

The population of parturients not able to directly communicate with the staff (Category 1) was used as a reference group in the analysis.

### 2.4 Ethical Aspects

The hospital research review board for Obstetrics, Gynecology and Pediatrics gave authorization to perform the study (decisions HUS/614/2023, 8 November 2023 and HUS/707/2025, 23 January 2025). A signed informed consent was waived based on Finnish research law (488/1999 and 552/2019). Patient identities were pseudonymized and all data were processed in a secure electronic environment designed for processing of health data for scientific purposes (Findata regulation 1/2020 given on 5 October 2020).

## 3 Results

### 3.1 The Parturients

During the study period there were 13,707 documented attempted vaginal deliveries. The parturients had 91 different primary languages stored in the patient journaling system. Of the parturients, 10,197 (74.4%) had Finnish or Swedish marked as their primary language (category III). Of the remaining parturients, 1416 (10.3% of the whole cohort) were marked to communicate without the need for an interpreter in Finnish, Swedish, or English while having a primary language other than Finnish or Swedish and formed the communication language category II. The remaining 2094 (15.3%) of the parturients with a communication language other than Finnish, Swedish, or English formed communication language Category I.

The distribution of the parturients into the different language groups according to geographical areas of their primary languages is shown in Table 1. In the communication language Category I, the five most spoken languages were Russian, Arabic, Somali, Estonian, and Albanian which combined accounted for 47% of the parturients within Category I. The geographic area of the primary language is associated with different allocation likelihood into language capability Categories I and II. The parturients with a primary language from the European Economic Area (EEA) countries or Sub‐Saharan Africa area were significantly more likely to communicate in Finnish, Swedish, or English (Category II) than parturients from other main geographic regions (Table 1).

### 3.2 Association of the Communication Language Category With Differences in Background and Labor Parameters

The directly communicating non‐primary language speaking parturients (Category II) were more often primiparous, had higher BMI, went into labor at an earlier gestational age, underwent induction of labor, and more frequently ended up in intrapartum cesarean delivery than indirectly communicating parturients (Category I). The parameters and their comparison in the language categories II and the primary Finnish or Swedish speaking population (Category III) against the indirectly communicating population (Category I) are shown in Table 2.

**TABLE 2:** Parturient and labor parameters' association with communication language in the Helsinki area delivery hospitals in 2022.

Parturient and labor parameters' association with communication language in the Helsinki area delivery hospitals in 2022					
Communication language category^a^	I	II		III	
*N*	2094	1416	*p* (vs. Category I)	10,197	*p* (vs. Category I)
Background factors					
Primiparous	1358 (64.9%)	975 (68.9%)	0.014	7239 (71.0%)	< 0.001
Age (year)	32.4 (4.84)	32.3 (4.88)	0.758	32.5 (4.91)	0.204
BMI (kg/m^2^)	24.8 (4.71)	25.5 (5.13)	< 0.001	25.1 (5.05)	0.014
Gestational age (weeks)	39.8 (1.70)	39.7 (2.08)	0.040	39.8 (1.66)	0.211
Preterm < 32 weeks	14 (0.7%)	18 (1.3%)	0.067	56 (0.6%)	0.523
Fear of childbirth diagnosis	150 (7.2%)	124 (8.8%)	0.084	1361 (13.3%)	< 0.001
Labor					
Induction	555 (26.5%)	437 (30.9%)	0.005	3043 (29.8%)	0.002
Oxytocin use (augmentation)	690 (33.0%)	498 (35.2%)	0.173	3650 (35.8%)	0.013
Labor outcome					
Episiotomy	190 (9.1%)	114 (8.1%)	0.291	995 (9.8%)	0.350
Spontaneous vaginal delivery	1601 (76.5%)	1042 (73.6%)	0.053	7653 (75.1%)	0.182
Instrumental vaginal delivery	227 (10.8%)	157 (11.1%)	0.818	1236 (12.1%)	0.104
Intrapartum cesarean delivery (CD)	266 (12.7%)	217 (15.3%)	0.027	1308 (12.8%)	0.912

*Note:* Data shown as *N* (percentage) within the indicated communication language category for categorial variables and mean (standard deviation) for continuous variables. *p* values calculated for intergroup comparison against Category I (chi‐square test for categorical data and independent sample *t*‐test for continuous variables).

^a^ I: Communicates in other language (than Finnish, Swedish, or English); II: Primary language other than Finnish or Swedish, communicates in Finnish, Swedish or English without documented need for interpreter; III: Primary language Finnish or Swedish.

### 3.3 Use of Neuraxial Analgesia in Different Communication Language Categories

**FIGURE 1:**
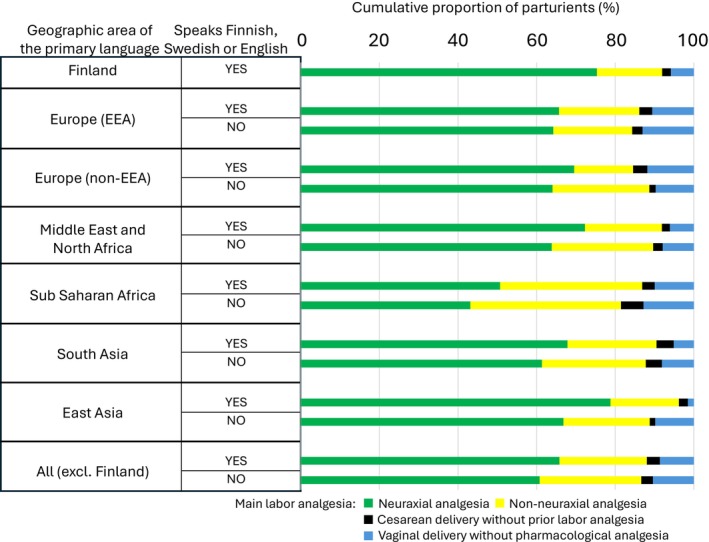
Cumulative use of labor analgesia in different geographic primary language groups. The proportion of parturients using neuraxial analgesia with or without any other analgesia are shown in green and the proportion of parturients using any combination of non‐neuraxial analgesia only are shown in yellow. Solid and blue bars show parturients that did not use any form of labor analgesia. Blue bars show vaginal delivery without pharmacological labor analgesia.

Neuraxial analgesia was used less frequently among parturients whose primary language was other than Finnish or Swedish compared to the population whose primary language was Finnish or Swedish (Figure 1). Neuraxial analgesia was used in 60.8%, 65.8%, and 75.3% of the deliveries in the communication language Categories I, II, and III, respectively (Table 3, Figure 1). The unadjusted odds ratio (OR) for neuraxial analgesia use in Category II vs. Category I was 1.242 (1.079–1.429). After adjustment for factors differentiating the communication language categories and associating with neuraxial analgesia use (geographic area of primary language, primiparity, BMI, diagnosed FOC, gestational age, induction of labor, oxytocin use, episiotomy use, and labor outcome), the parturients in category II that could directly communicate with the staff were more likely to use neuraxial analgesia compared to those parturients that could not communicate directly (aOR 1.283 [1.093–1.506]).

**TABLE 3:** Communication language category‐association with labor analgesia use in the Helsinki area delivery hospitals in 2022.

Communication language category^a^	I	II		III	
*N*	2094	1416	*p*	10,197	*p*
Used labor analgesia methods					
Systemic opioid analgesia	448 (21.4%)	336 (23.7%)	0.103	2486 (24.4%)	0.699
Nitrous oxide	1329 (63.5%)	895 (63.2%)	0.875	7078 (69.4%)	< 0.001
Paracervical block	184 (8.8%)	119 (8.4%)	0.692	748 (7.3%)	0.013
Pudendal block	258 (12.3%)	176 (12.4%)	0.924	1770 (17.4%)	< 0.001
Neuraxial analgesia^b^	1273 (60.8%)	932 (65.8%)	0.003	7683 (75.3%)	< 0.001
Delivery without pharmacological pain relief	280 (13.4%)	169 (11.9%)	0.211	820 (8.0%)	< 0.001
Vaginal delivery without pharmacological pain relief	218 (10.4%)	122 (8.6%)	0.078	595 (5.8%)	< 0.001

^a^ I: Communicates in other language (than Finnish, Swedish or English); II: Primary language other than Finnish or Swedish, communicates in Finnish, Swedish or English without documented need for interpreter; III: Primary language Finnish or Swedish.

^b^ Includes single shot spinal, epidural and combined spinal‐epidural analgesia placed during labor for labor analgesia.

The geographic area represented by the primary language is associated with neuraxial analgesia use (Figure 1). In the multivariate analysis, parturients with a primary language from sub–Saharan Africa were less likely to use neuraxial analgesia while the other cofactors associated with neuraxial analgesia use were similar to those observed in the primary Finnish or Swedish speaking population: primiparity, advanced gestational age, diagnosed FOC, use of oxytocin, and labor ending up in episiotomy, instrumental vaginal delivery or operative delivery. The use of other labor analgesia forms showed significant associations with neuraxial analgesia use with systemic opioids or nitrous oxide use increasing and paracervical block decreasing the concomitant neuraxial analgesia use (see Supporting Information).

### 3.4 Use of Other Forms of Labor Analgesia and Delivery Without Pharmacological Pain Relief

There were no differences between the communicating language Categories I and II in the use of systemic opioid analgesia (oxycodone), nitrous oxide, paracervical or pudendal blocks. The absolute use rate of labor analgesia forms in the different communicating language categories is summarized in Table 3. While no difference is seen between communicating language Categories I and II in any particular labor analgesia type besides neuraxial analgesia, the combined use of various pharmacological non‐neuraxial analgesia is less common among directly communicating parturients compared to non‐directly communicating parturients (Category II vs. Category I; aOR 0.789 [0.654–0.951]).

Except for systemic opioids and paracervical blocks, the different labor analgesia types were used less frequently among parturients with a primary language other than Finnish or Swedish compared to those parturients whose primary language was Finnish or Swedish. The only notable exception is the use of paracervical blocks, which were more commonly used in the non‐Finnish or Swedish primary language speaking population compared to the primary Finnish or Swedish speakers (Table 3). Of the primary Finnish or Swedish speaking parturients who had paracervical block, 70% had also neuraxial analgesia, compared to 51% in the population whose primary language was other than Finnish or Swedish.

Among foreign primary language speakers (Categories I and II) there was no difference between those parturients that could communicate indirectly or directly in the odds of delivering without pharmacological pain relief by any delivery mode (aOR 0.815 [0.652–1.019]) or vaginally (aOR 0.825 [0.645–1.055]).

### 3.5 Association of Communication Language With the Documentation of Birthing Experience Score and Analgesia Satisfaction

**FIGURE 2:**
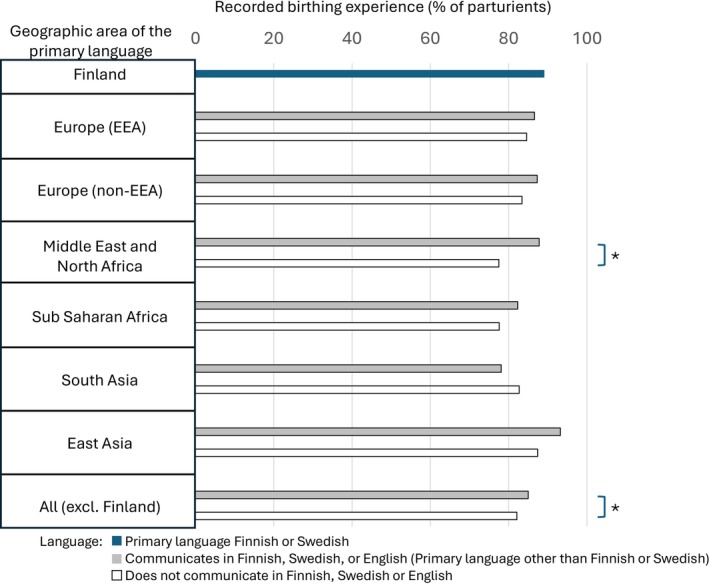
The rate of recording of the birthing experience after the delivery in different parturient populations by the geographical area of the primary language. *p < 0.05, chi‐square test between indicated groups.

The birthing experience score was documented for 89.1% of the primary Finnish or Swedish speaking population (Category III) and 85.0% of the parturients who could communicate directly (Category II), while the documentation rate was 82.1% in the indirectly communicating subpopulation (Category I). Thus, being able to directly communicate with the care providers increased the likelihood of having the birthing experience score documented (univariate OR 1.232 [1.025–1.480]). Parturients with a primary language from the Middle East or Sub‐Saharan Africa area who could not communicate directly were least likely to have their birthing experience documented (Figure 2).

**FIGURE 3:**
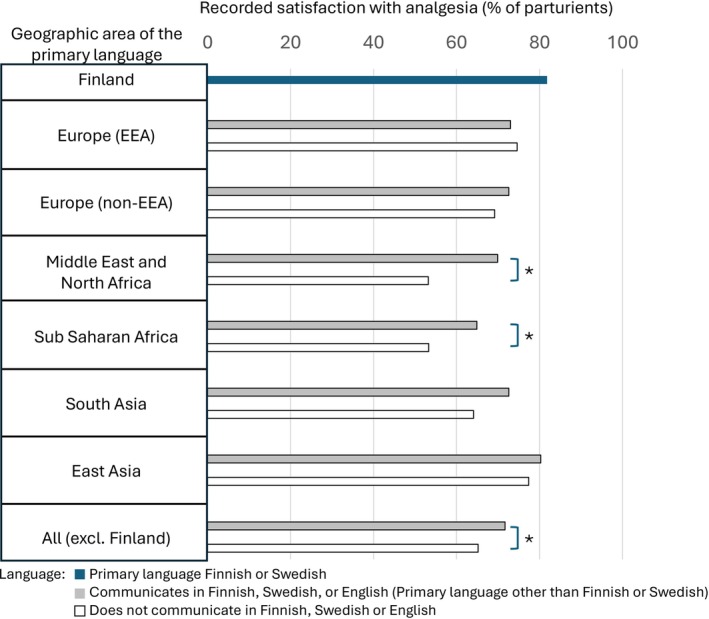
The rate of recording of satisfaction on labor analgesia in different parturient populations by geographic area of the parturient's primary language. *p < 0.05, chi‐square test between indicated groups.

The maternal satisfaction score on analgesia was recorded for 81.8% of the primary Finnish or Swedish speakers (Category III) that used pharmacological labor analgesia. The documentation level declined with decreasing communicating language capability and was 71.7% in the language Category II and 65.2% in Category I (Figure 3). Ability to communicate directly increased the likelihood of having the satisfaction score documented (univariate OR 1.351 [1.155–1.580]) in communication language categories I and II. The lowest recording rate was seen among parturients who spoke languages from the Middle East and Sub‐Saharan Africa area and did not communicate directly (Figure 3). The recording rate increased significantly if the parturient could directly communicate with the staff (Figure 3).

The number of parturients reporting full satisfaction on analgesia was 638 and 472 in language Categories I and II, respectively. This accounts for 53.9% and 52.8% of the documented values in these communication language categories that used some form of pharmacological pain relief, respectively. The corresponding figure among the primary Finnish or Swedish speaking population was 34.5%. Use of neuraxial analgesia was associated with increased odds of reporting full satisfaction on analgesia in all communication language categories (Category I: 1.353 [1.047–1.749]; Category II: 1.524 [1.117–2.080]; Category III: 1.535 [1.344–1.754]).

Full satisfaction on analgesia was reported by 35.2%, 37.9%, and 28.3% of the whole parturient population using pharmacological pain relief in language Categories I, II, and III, respectively. The use of neuraxial analgesia increased the OR of reporting full satisfaction on analgesia in all communication language categories even if the missing figures on satisfaction are considered to represent less than complete satisfaction on analgesia (Category I OR: 1.505 [1.210–1.870]; Category II OR: 1.604 [1.219–2.111]; Category III OR: 1.654 [1.456–1.879]).

## 4 Discussion

Our study shows that among parturients whose primary language is other than Finnish or Swedish, the official languages in Finland, the ability to communicate directly with the healthcare staff is associated with an increased likelihood of neuraxial labor analgesia use compared to parturients who cannot directly communicate with the staff. Such ability appears to play a significant role in neuraxial analgesia use irrespective of the primary language among parturients who speak a foreign primary language. However, the geographical area of the primary language appears to associate more strongly with the choice of using neuraxial analgesia, suggesting that cultural aspects may play a larger role than direct communication capability in the labor analgesia choice.

The language profile of the parturients seen in our cohort follows closely the reported primary languages spoken in the capital region of Finland by female inhabitants aged 18–40. According to the central population registry, 26% of all parturients that gave birth in the greater Helsinki area in 2022 were either born abroad or had both of their parents born abroad [2]. Due to the relatively short history of immigration to Finland, only 0.8% of all parturients were themselves born in Finland but had both parents born abroad [2]. Therefore, nearly all parturients that have a primary language other than Finnish or Swedish are themselves first‐degree immigrants. Along with their languages, the parturients with a foreign primary language may bring with them their cultural beliefs and expectations on labor pain and its treatment. Meeting these expectations in a system that relies rather strongly on neuraxial analgesia use may prove challenging, particularly since some minority language‐speaking parturient groups may hold strong opinions on the lack of safety of neuraxial analgesia [7, 8].

The primary outcome of this study is the association of capability to communicate in Finnish, Swedish or English on the use rate of neuraxial analgesia among parturients whose primary language is not Finnish or Swedish. English is widely taught and spoken in Finland and basically all Finnish healthcare staff can communicate in English. Our results indicate that the use rate of neuraxial analgesia increases when the parturients can directly communicate with staff, also after adjustment for potential cofactors associated with neuraxial analgesia use. Compared to the indirectly communicating parturients, the parturients with a foreign primary language that can directly communicate with the healthcare staff undergo induction of labor more often and end up delivering at a slightly earlier gestational age (Table 2). This may implicate better adherence to antenatal maternity care.

Previous studies from Norway and Sweden have identified several factors that can associate with the choice of using neuraxial analgesia in labor [3, 4, 5, 9]. The duration of stay in the host country, higher education level, and having a host country‐born partner all appear to increase the likelihood of neuraxial analgesia use [3, 4, 5, 9]. The prior large‐scale population studies completed by matching national birth registries with population databases provide valuable information on the potential associations between the background variables and the actual labor analgesia use. However, these studies cannot address the language capabilities of the parturient, which may have vital associations with the actual choice of labor analgesia. Also, the use of other forms of labor analgesia is often overlooked. The factors shown to increase the likelihood of neuraxial analgesia use, such as receiving country‐born companions or longer stay in the host country, may serve as surrogates for a higher chance of being able to directly communicate with the healthcare personnel.

The secondary outcome of our study is the overall use of pharmacological labor analgesia, which is addressed by assessing the rate of delivery without any pharmacological pain relief. It was noted that parturients who ended up in intrapartum cesarean delivery did so at an earlier stage than those who used pharmacological pain relief and in this sense for some the labor pain relief was replaced by neuraxial anesthesia for cesarean delivery. Therefore, the actual assessment outcome was set as vaginal delivery without pharmacological pain relief (Table 4, Figure 1). While vaginal delivery without pharmacological analgesia was more prevalent among those parturients whose primary language was other than Finnish or Swedish compared to the primary Finnish or Swedish speaking population (Table 3), there was no statistically significant difference between parturients that could or could not communicate directly with the staff (Table 4). This further emphasizes the association of cultural or other factors such as educational level or previous birthing experiences rather than language capabilities on the use of pharmacological labor analgesia.

**TABLE 4:** Association of communication language with labor analgesia use among parturients whose primary language is other than Finnish or Swedish.

Labor analgesia type	Parturients that communicate in Finnish, Swedish, or English vs. those that cannot communicate directly with the healthcare staff			
	OR (95% CI)	*p*	aOR (95% CI)^b^	*p*
Neuraxial analgesia	1.242 (1.079–1.429)	0.003	1.283 (1.093–1.506)	0.002
Non‐neuraxial analgesia	0.821 (0.701–0.963)	0.015	0.789 (0.654–0.951)	0.013
No pharmacological analgesia	0.878 (0.716–1.077)	0.212	0.815 (0.652–1.019)	0.073
No pharmacological analgesia^a^	0.811 (0.643–1.024)	0.078	0.825 (0.645–1.055)	0.126

^a^ OR and aOR calculated for outcome: vaginal delivery without pharmacological analgesia.

^b^ Adjusted for geographical area of the primary language, primiparity, gestational age, fear of childbirth diagnosis, induction of labor, oxytocin use during delivery, episiotomy, outcome of labor (spontaneous vaginal, instrumental vaginal, intrapartum cesarean delivery except in ^a^), use of other labor analgesia (systemic opioid, paracervical block), delivery unit.

Regarding the choice of individual labor analgesia types, the only difference between the directly communicating and indirectly communicating primarily non‐Finnish or Swedish speaking groups is seen in the use of neuraxial analgesia (Table 3). Another notable difference is the more common use of paracervical block in both the directly and indirectly communicating parturient population compared to the primary Finnish or Swedish speaking population (Table 3). This likely reflects the institutional practice where paracervical block is used mainly to treat labor pain in parturients who wish to avoid neuraxial analgesia and this approach appears to work relatively well among parturients whose primary language is not Finnish or Swedish compared to the primary Finnish or Swedish speaking population. The use rate of only non‐neuraxial analgesia is lower among parturients with direct communication capability compared to indirectly communicating parturients, also after adjustment for parturient and labor related cofactors (Table 4). This suggests that direct communication capability is associated with a shift from non‐neuraxial analgesia techniques to neuraxial techniques and likely reflects some form of shared decision‐making with the parturient.

In Finland no formal written consent is required for the neuraxial analgesia to be delivered and in the government provided public healthcare it has been shown that socioeconomical factors do not influence the neuraxial analgesia use [10]. The decision on labor analgesia use is made between the parturient and the midwife caring for her. If the two cannot communicate directly, some information may be lost or modified in between the parties involved in the decision‐making process. Nowadays, there exist translation services by telephone in several languages making communication easier. In cases where an official translator is unavailable, communication relies on the language skills of a friend or relative, sign language, drawing, or internet‐based translation programs. Also, since 2022, when the parturients in this cohort delivered, the spectrum of primary languages spoken by the healthcare professionals in the maternity hospitals has widened reflecting the overall structural change in the local population. Such availability of healthcare professionals that speak the same primary language as the parturient has significantly improved the quality of communication with the parturients that could otherwise not communicate directly.

There are apparent cultural differences in the immigrant parturient populations relating to the use of labor analgesia and particularly neuraxial analgesia. A prior study from Sweden showed that immigrant parturient's country of origin had a significant association with neuraxial analgesia use with a higher use rate among the Finnish immigrants and substantially lower use in parturients from the former Yugoslavian area, the Middle East and Somalia [3]. In agreement with our results, the use of neuraxial analgesia was least frequent in parturients whose primary language was Somali, which constitutes most of our Sub‐Saharan Africa language area subgroup. Meanwhile, the overall neuraxial use rate in the prior Swedish study was 40.1% and the study only included primiparous parturients. The study did not address language proficiency but showed that if the parturient's partner was born in Sweden, the odds for neuraxial analgesia use increased, which was also the case if the parturient had lived for a longer time in Sweden [3]. Similar association with lower epidural analgesia use among sub‐Saharan area immigrants has been shown in a study from Norway, which also showed that a higher education level and longer residence time in Norway increased the likelihood of having epidural analgesia [5].

The cultural attitudes towards neuraxial analgesia are likely to persist even when the parturient communicates in Finnish or Swedish. Many of the parturients whose primary language is spoken in the Sub‐Saharan area in our cohort speak Somali as their primary language and many of them may have moved to Finland in the early 1990's when they were very young. Despite this, the use of neuraxial analgesia is much less frequent while many of them speak native level Finnish. The relatively better communication capacity of the sub‐Saharan area immigrants is evident in Table 1 which shows that a higher proportion of Sub‐Saharan area primary language speakers can communicate in Finnish, Swedish or English compared to South Asian, Middle Eastern or East Asian language speakers who represent as a group much more recent immigration to Finland. Still, the neuraxial use rate in non‐directly communicating immigrants speaking languages from Europe and Asia exceeds the neuraxial use rate among Sub‐Saharan African area primary language speakers who are able to communicate directly. Thus, it is apparent that the background culture of the parturient plays a major role in the use of neuraxial analgesia as was also shown in the prior Scandinavian studies [3, 4, 5, 9]. On the other hand, being able to directly communicate with the health care staff increases the likelihood of neuraxial analgesia use in the whole population whose primary language is not Finnish or Swedish irrespective of the geographic area of the primary language (Figure 1; Table 4).

A substantial proportion (47%) of those parturients whose primary language was other than Finnish or Swedish using non‐neuraxial analgesia reported full satisfaction on analgesia as opposed to 27% in the primary Finnish or Swedish speaking population. Thus, our data suggests that a high level of satisfaction on analgesia is attainable also without neuraxial analgesia within the parturients whose primary language is other than Finnish or Swedish. However, the proportion of parturients whose satisfaction score is not recorded increases alarmingly among minority language groups, even when they have been categorized as being able to communicate with the staff (Figure 3) and can report overall birthing experience score (Figure 2). Due to the lower reporting frequency among language subgroups, these figures should be considered with some caution. Neuraxial analgesia use was found to associate with higher reporting frequency for analgesia satisfaction and may partially explain the reporting bias. However, even if the percentages of fully satisfied parturients are calculated by assuming that none of the non‐responders were fully satisfied with their analgesia, the results show that parturients whose primary language is other than Finnish or Swedish are more satisfied with their labor analgesia and this satisfaction is further increased when neuraxial analgesia is used. This phenomenon may indicate higher expectations for labor analgesia among primary Finnish or Swedish speaking population compared to minority language groups as primiparity, induction of labor, and FOC have been shown to associate with reduced parturient satisfaction with labor analgesia in our study population [11]. Fear of childbirth is screened during antenatal visits in primary maternity care and parturients with suspected FOC are referred for consultation and treatment in the delivery hospital where the diagnosis is set. Experiencing FOC often includes fear of pain, and therefore adequate labor analgesia has a significant effect on the overall birth experience [12, 13].

As the type, or even existence, of labor analgesia may vary considerably in various parts of the world, parturients whose primary language is other than Finnish or Swedish may not always be aware of all possible pain relief methods available in Finnish delivery hospitals [7]. These should be discussed already in primary maternity care, which could even alleviate FOC knowing that there exists efficient pain relief that can be asked for.

Since being able to communicate with the caretakers facilitates shared decision making and appears to increase the use of neuraxial labor analgesia, providing all parturients with better information regarding the labor analgesia options in their own language could promote the use of pharmacological labor analgesia, including neuraxial analgesia. A freely available source of information (www.laborpains.org) is in use at the labor wards in the Helsinki area and the information on labor analgesia options would be available for 65% of the parturients in the language Category I, who would not be able to directly communicate with the healthcare staff.

It is important to note that there are some limitations to our study due to the retrospective nature of it. The associations seen between the communication language category and labor analgesia use may not indicate direct causality as there may be some uncontrolled confounders such as educational level, cultural background factors as well as socio‐economic factors that were not controlled for. The geographic area of the primary language was used to adjust for the differences in the likelihood of allocation into communication language categories I and II and may also provide partial adjustment for the underlying cultural differences. The retrospective nature of this study may also affect the accuracy of the information recorded. On the other hand, most of the information is mandatory to be filled in the system and undergoes validation before being sent to the national statistics authority for birth recording. Being able to speak Finnish, Swedish or English may indicate longer stay in Finland prior to delivery, and therefore possibly better integration into the society or alternatively higher education level of the parturient. Neither the information on the duration of stay in Finland nor the education level of the parturient is documented in the patient journaling system and therefore this information was not available. The information on the quality and counseling provided by the midwives during labor, as well as the outcome or possible labor analgesia use in prior deliveries was also not available.

The results of our study should be generalizable to other publicly funded health care systems serving mixed immigrant and native‐born populations with different communication language skills. However, the equity in access to care may limit generalization. The Finnish healthcare system aims to provide equitable care, and particularly maternity care, to all women giving birth irrespective of the financial, social, or immigration status. Thus, the results may not be generalizable to systems providing less equitable access to maternity care. The maternity hospitals where the deliveries took place provide a neuraxial analgesia service at all times in compliance with the Finnish law governing the resources for maternity hospitals and any available labor analgesia is provided without additional cost barriers to the parturient.

In conclusion, parturients who can directly communicate with the healthcare staff are more likely to use neuraxial analgesia compared to indirectly communicating parturients, who are more likely to use other non‐neuraxial labor analgesia types. The communication language used by the parturient does not seem to be associated with the rate of vaginal deliveries without pharmacological pain relief. There are significant differences in neuraxial analgesia use rates depending on the geographical area of the primary language of the parturient. The lack of a common communication language associates with a poorer documentation rate of maternal satisfaction on analgesia. Therefore, we should not only focus on providing adequate information on the available labor analgesia options but also acknowledge the right to equal care for all parturients by documenting the birth experience scores for all parturients.

## Author Contributions

**Luisa Pirsko:** conceptualization, data analysis, visualization, edited the manuscript draft, read and approved the final version of the manuscript. **Anna L. U. Väänänen:** collection of the data, data analysis, visualization, editing the final version of the manuscript, approved the final version of the manuscript. **Riina Jernman:** conceptualization, collection of the data, analysis of the data, read and approved the final version of the manuscript. **Antti Väänänen:** conceptualization, collection of the data, analysis of the data, wrote the first version of the manuscript, read and approved the final version of the manuscript, project management.

## Funding

Antti Väänänen received funding from the Finnish Medical Foundation (Suomen lääketieteen säätiö) for this study (grant number 5989).

## Ethics Statement

Institutional research board approved the study.

## Conflicts of Interest

The authors declare no conflicts of interest.

## Supporting information


**Data S1:** aas70248‐sup‐0001‐Supinfo.xlsx.

## Data Availability

The data that support the findings of this study are available from HelsinkiUniversity Central Hospital. Restrictions apply to the availabilityof these data, which were used under license for this study. Data areavailable from the author(s) with the permission of Helsinki UniversityCentral Hospital.
